# Can a Novel Device with Pure Dry Air Increase the Shear Bond Strength of Dental Composites to Dentin? An Experimental Study

**DOI:** 10.3390/dj12060160

**Published:** 2024-05-24

**Authors:** Khalil Kharma, Louis Hardan, Cynthia Kassis, Bogdan Dimitriu, Ryan Harouny, Nadim Z. Baba, Rim Bourgi, Carina Mehanna Zogheib

**Affiliations:** 1 Department of Restorative Dentistry, School of Dentistry, Saint-Joseph University, Beirut 1107 2180, Lebanon; khalil.kharma@usj.edu.lb (K.K.); cynthia.kassis@usj.edu.lb (C.K.); ryaneliott.harouny@net.usj.edu.lb (R.H.); rim.bourgi@net.usj.edu.lb (R.B.); carina.mehannazogheib@usj.edu.lb (C.M.Z.); 2 Department of Endodontics, Deputy Dean, Faculty of Dental Medicine “Carol Davila” University, 020021 Bucharest, Romania; 3 Craniofacial Research Laboratory, Division of Biomaterials, School of Dentistry, Saint-Joseph University, Beirut 1107 2180, Lebanon; 4 Advanced Dental Education Program in Implant Dentistry, School of Dentistry, Loma Linda University, Loma Linda, CA 92350, USA; nbaba@llu.edu; 5 Department of Biomaterials and Bioengineering, INSERM UMR_S 1121, University of Strasbourg, 67000 Strasbourg, France

**Keywords:** adhesive, bond strength, dentin, humidity, universal adhesive

## Abstract

Modern conservative dentistry is taking the lead in daily clinical practice and is relying on adhesion. Whether it is a simple composite, ceramic inlays, onlays, veneers or crowns, the common factor for a successful outcome is a good bonding of these elements to dental structures. Thus, the purpose of this study was to evaluate the bond strength of resin composite to dentin when using a new device, the DENTIPURE KM™ (KM, Beirut, Lebanon), which provides a pure air flow, free of any contaminants and without humidity, when compared to other dental equipment. One hundred and eighty extracted human molars were equally divided into three groups according to the device used, the DENTIPURE KM™ (KM, Beirut, Lebanon), the KAVO™ (ESTETICA E30/E70/E80 Vision, KAVO, Biberach, Germany), or the ADEC™ (A-dec Performer 200, Newberg, OR, USA). The shear bond strength (SBS) was evaluated after 24 h of storage in distilled water on a universal testing machine. Statistical analysis was set with a level of significance at *p* ≤ 0.05. The results revealed that significantly different bond strength was imparted by the DENTIPURE KM™ device and the ADEC™ dental unit (*p* = 0.042). In conclusion, while the DENTIPURE KM™ device shows promise in providing contaminant-free air during bonding, its impact on dentin bond strength compared to devices like the KAVO™ appears minimal. Further research is needed to fully assess its potential in enhancing dentinal adhesion procedures.

## 1. Introduction

Dental adhesive technology is in continuous development to constantly improve the shear bond strength (SBS) of resin composites to dentinal structure for a better adhesion of direct composites, in addition to full and partial indirect ceramic restorations. Several generations of adhesives have been introduced to simplify the clinical procedures and to obtain a predictable and a reproducible clinical outcome [1,2]. However, bonding to dentin is challenging. This can be explained by the nature of the dentin substrate, which can jeopardize long-term treatment success. The key factor for failure of the resin composite restoration is nanoleakage, which causes hypersensitivity, secondary caries, and long-term loss of the restoration [3].

Two approaches can be utilized for dentin adhesion: etch-and-rinse (ER) or self-etch (SE) bonding mode. The latest generation is the universal adhesives (UAs) that can be used in both ER and SE modes. The main feature of this generation of adhesive is that it contains both hydrophilic and hydrophobic components mixed in one bottle [4]. It is important to emphasize that the hybrid layer (HL) is considered as the fundamental diffusion layer that fulfills the occurrence of micromechanical retention, thus leading to a successful dentin adhesion [3].

Dental adhesives should initially be hydrophilic to properly wet moist dentin. They should also transform after polymerization into a hydrophobic state, to prevent water sorption and degradation of the bonded surface. The mechanism of ER can be described as a diffusion-based micromechanical interlocking. The phosphoric acid applied on the dental substrates can produce deep pits in the hydroxyapatite (Hap)-rich enamel and can demineralize the dentin up to 5–6 μm of depth, thus exposing the Hap-free collagen network considered as an essential part of the HL structure. Monomers of the UAs diffuse into the created micro-etched pits of the enamel to form micro- and macro-tags, and into the exposed collagen–fibril network of the dentin, to form a 3–5 μm HL, similar to the one obtained with the use of conventional ER adhesives [5].

Dental UAs contain organic solvents (ethanol or acetone) to facilitate monomer infiltration into the humid dentin substrate. Although water and organic solvents are essential components of UAs, they should be completely evaporated by consistent air flow. The evaporation of water and organic solvents prevents their interference with the polymerization process of the monomers composing the adhesives, resulting in a weaker bond to dentin. Excess solvent in the polymerized adhesive may result in a porous structure at the adhesive/dentin interface [6]. Hence, the process of solvent evaporation is crucial in order to enhance the polymerization reaction of resin monomers. This can be achieved by reducing the distance between the monomers, thus increasing the degree of conversion [7]. Adequate agitation of the adhesive and blowing air with the use of an air/water syringe are often used to evaporate the solvent. This procedure is technique-sensitive and seems to be difficult to achieve in practice. Several techniques have been discussed to improve the strength of resin composite bonds to dentin. On one hand, it was previously reported that in some cases the solvents need up to 20 min to completely evaporate [8]. On the other hand, a study conducted by Issis et al. showed that increasing the evaporating time of the solvent in the UAs in ER mode from 5 s to 25 s resulted in higher SBS of the adhesive [9].

Furthermore, a systematic review and meta-analysis on the effect of a warm airstream in solvent evaporation on the dentinal bond strength of adhesive systems found that warm air facilitates the evaporation of the solvent, mainly water and ethanol-based solvent, thus increasing the bond strength of the adhesive [10].

To date there are no evidence-based studies that assessed the quality of the air flow generated by multifunction syringes in dental units. Due to its mechanism, an air/water syringe often generates humidity. To check the level of humidity and water in the air syringe, it is recommended to blow air from the syringe into a receptacle containing anhydrous copper sulfate, which turns blue when in contact with humidity and water [11]. When evaporating the solvent, excess humidity can lead to a decrease in the bond strength of dental adhesives [12]. Accordingly, the aim of the current research was to assess whether the use of an innovative pure air device (DENTIPURE KM™, KM, Beirut, Lebanon) enhanced the bond strength of a universal adhesive to dentin. Hence, the null hypothesis tested was that there would be no difference in the SBS to dentin when using this new device compared to other dental units.

## 2. Materials and Methods

### 2.1. Study Design and Specimen Fabrication

To determine the sample size, a power analysis was performed using G*Power software for analysis of variance (ANOVA) (fixed, omnibus, one way), considering a power of 80%, alpha error of 5%, and an effect size equal to 0.25. The minimum sample size required was 159, hence about 54 teeth per group. Accordingly, intact fresh extracted human molars (n = 180) were selected randomly for this study. The teeth were fixed in 10% formalin for two weeks [13]. Then, they were immersed in distilled water with anhydrous sodium in the following proportions: 0.2 g of anhydrous sodium was diluted in 100 mL of distilled water. The water in which the teeth were bathed was changed weekly to preserve the physiological properties of the teeth and to maintain adequate dentin physiological properties.

Upon the approval of the research ethical committee of the Saint-Joseph University of Beirut, Lebanon (USJ-2020-17), these teeth were fixed by their roots in a hard yellow dental gypsum base (V DENTAL™, Bielawa, Poland) while keeping their dental crown free. Next, using a low-speed precision cutting machine (EXAKT Vertriebs GmbH, Norderstedt, Germany) with constant irrigation, a homogeneous cut in the mesio-distal direction was made to a depth of 2 mm to reach the dentin. Polishing was performed on the entire dentin surface by 600-grit silicon carbide papers (Uxcell™, Hong Kong, China) for 1 min to create a uniform surface under water irrigation. This procedure was performed by a single operator to limit preparation bias. Then, the teeth were randomly divided into 3 different groups to have n = 60 teeth in each group.

In the first group (control group), the A-DEC type dental chair (A-dec™ Performer 200, Newberg, OR, USA) was used to perform the drying and rinsing steps. This group presented features including a 2.5-bar pressure, full air filtration, and air ambient temperature. A second group was prepared using a dental chair equipped with an automatic disinfection system (ESTETICA™ E30/E70/E80 Vision, KAVO, Biberach, Germany) used to perform the drying and rinsing steps. Finally, in the third group, samples were prepared using a new device (DENTIPURE KM™, KM, Beirut, Lebanon). This device was specified for obtaining sterile water and compressed medical air. In addition, this device was developed to assess the humidity issue in dental adhesion. Indeed, this device was designed in a way that the air duct is separated from the sterile water line, thus eliminating the possibility of mixing air and humidity. Additionally, the air source comes from a compressed bottle and not from the regular compressor of the dental unit, which may also contribute to the contamination of the air syringe by humidity and other impurities when using the traditional multifunction syringe.

This device is also capable of supplying sterile water under pressure as well as a decontaminating agent such as chlorhexidine. This device comprises a cylinder of medical compressed air with a flow regulator valve provided by the company Air Liquide and supplies air composed of 79% nitrogen and 21% oxygen. The compressed air cylinder is connected to an air syringe by a 1 × 3 mm autoclavable silicone tube (REALME™, Amiens, France). Also included are a 1 L autoclavable sterile water bottle (DURAN ™, Colombes, France) and a bottle containing chlorhexidine or another 1 L disinfecting product (DURAN ™, Colombes, France) (Table 1).

For dentin bonding, composite cylinders were elaborated using a special device (Ultradent™, South Jordan, UT, USA). The latter fixed the gypsum base in which the tooth had been introduced. At the level of the crown, a plate containing a cylindrical funnel was used to inject the composite. The particularity of this bonding jig is to make it possible to obtain composite cylinders of uniform dimensions (internal diameter of 2.38 mm and height of 2.15 mm) on the prepared teeth.

The ER strategy was adopted for the filling of composite cylinders as follows: dentin etching was performed for 15 s with 40% orthophosphoric acid (ONYX™, CENTRIX, Shelton, CT, USA), followed by rinsing of the orthophosphoric acid for 15 s and drying of the dentin until a slightly moist surface was obtained. The next step was the application of the adhesive (Prime & Bond Universal (PBU), DENTSPLY, Charlotte, NC, USA) for 20 s with a micro-brush in a continuous and uniform motion (Table 2).

Following adhesive application, air was blown on the surface for 10 s to ensure the spreading of the adhesive as well as the evaporation of the solvent. Polymerization of the adhesive was carried out for 20 s using a light-emitting diode (LED) multiwave light-curing unit, CuringPen-E (Eighteeth, Changzhou, China), calibrated at 1000 mW/cm^2^. The injection of a bulk-fill flowable composite (SDR+, DENTSPLY, Charlotte, NC, USA) was achieved through the funnel, then polymerization was performed for 40 s using the same light-curing device. All restorations were stored at 37 °C in distilled water, in the incubator, for 24 h before the SBS test.

### 2.2. Bond Strength Test

**Figure 1:**
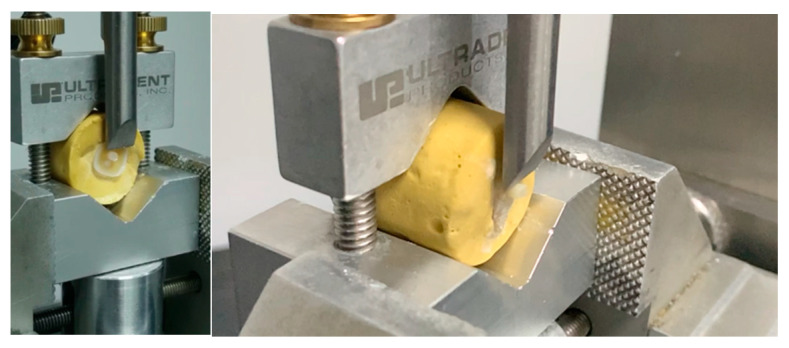
Shear bond strength testing on a universal testing machine.

After the adhesion procedure, the SBS was performed on a universal testing machine (EXAKT Vertriebs GmbH, Norderstedt, Germany) with a load cell of 500 N. Teeth were placed in a horizontal position and fixed by their gypsum base, thereby releasing the coronal part restored by the composite cylinder (Figure 1).

According to ISO/TS 11405:2015 [14], a crosshead speed of 1 mm/min was applied to each sample using a sharp blade until the bond between the composite cylinder and the dentin was broken. The values in MPa were noted for each specimen following the formula of R (MPa) = F (Newtons)/A(mm^2^); the bond strength was linked to the R, the failure force was correlated to the F, and the bonding area corresponded to the A.

### 2.3. Failure Mode Analysis

Following the SBS tests, all of the failed specimens were observed with a stereoscopic microscope (Olympus CX41, Shinjuku, Japan) at 40x magnification to determine the failure modes. Failure modes were divided into adhesive, cohesive in resin, cohesive in dentin, and mixed failure.

**Figure 2:**
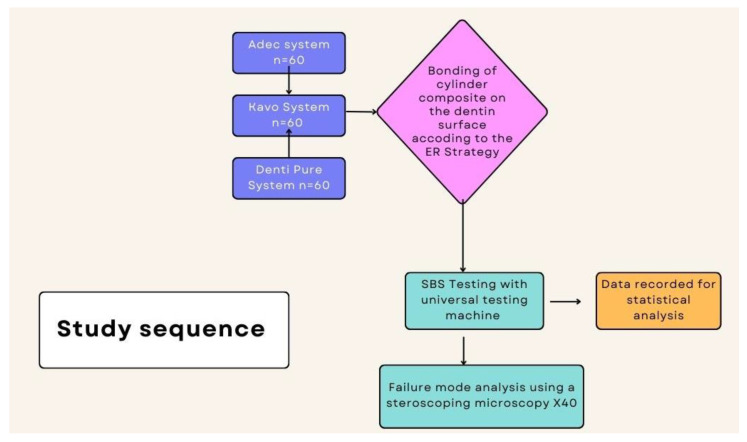
Study sequence.

The study sequence is summarized in the following figure (Figure 2).

### 2.4. Statistical Analyses

IBM SPSS Statistics (version 27) was used for statistical analysis. The level of significance was set at *p* ≤ 0.05. Kolmogorov–Smirnov tests were applied to assess the normality distribution of continuous variables. The primary outcome variable of the study was the SBS in MPa. One-way ANOVA followed by the Tukey (HSD) post hoc test were used to compare the mean SBS among groups.

## 3. Results

### 3.1. Bond Strength Test

**Figure 3:**
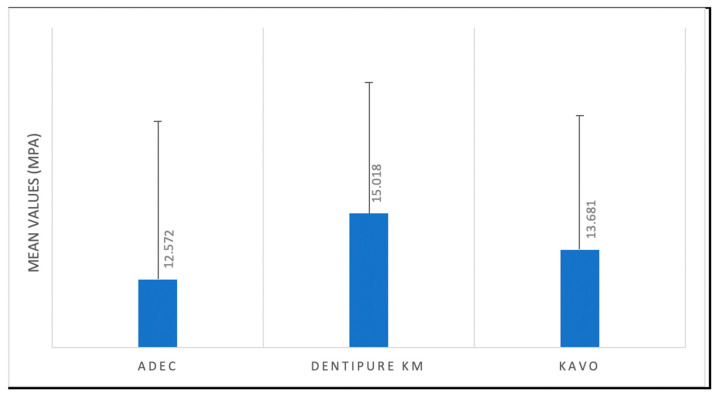
Mean shear bond strength in different groups.

The mean and standard deviation (SD) of the SBS (MPa) of the bonding tests are displayed in the following table (Table 3 and Figure 3).

The mean results were highest for the DENTIPURE KM™ group (15.018 MPa), intermediate for the KAVO group (13.681 MPa), and the lowest for the ADEC group (12.572 MPa). Further, bond strength was significantly different between samples tested with the DENTIPURE KM™ device and those tested with the ADEC™ dental unit (*p* = 0.042). According to Tukey’s multiple comparisons, the difference between the mean bond strength achieved via the KAVO and the of the other devices (Table 4) was not significant.

### 3.2. Failure Mode Analysis

**Figure 4:**
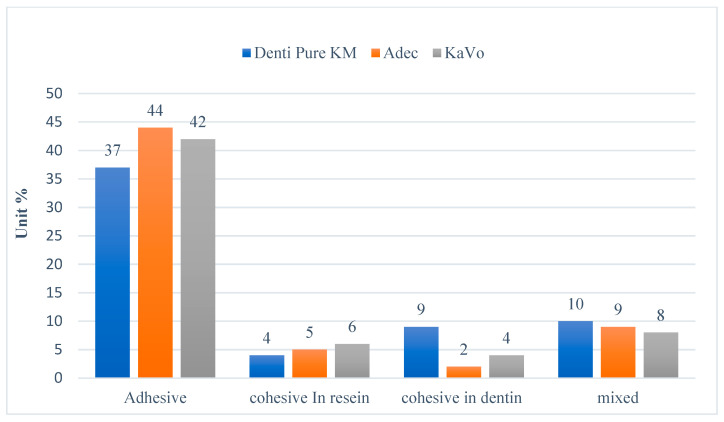
Failure mode analysis of the tested dentin specimens.

**Figure 5:**
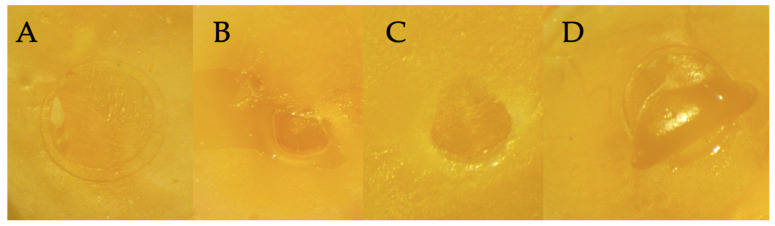
Different types of failure modes. (**A**) Cohesive in resin; (**B**) cohesive in dentin; (**C**) adhesive; (**D**) mixed.

The failure mode results are shown in Figure 4 and Figure 5.

Adhesive failure mode was dominant for dentin specimens tested regardless of the device used. Indeed, with the Dentipure KM device, 61.6% of the samples showed adhesive failures. With the ADEC system, 73.3% showed adhesive failures, and 70% failed with KAVO system.

## 4. Discussion

Composite resins and their adhesives have been the center of interest of researchers in recent decades. This can be explained by a growing interest and more demand from patients to undergo aesthetic direct and indirect restorations, which require a good adhesion to enamel and dentin because composites, unlike amalgam, do not require mechanical retention [15,16,17]. No previous study has focused on the air and water mechanism used by practitioners to bond composite restorations. The results obtained in this research showed that the adhesion to dentin was affected by the type of the device or equipment used for the drying and rinsing steps. Accordingly, the first null hypothesis tested was rejected.

Composite resin restorations require a certain skill from the practitioner and a perfect knowledge of the mechanism of action of the adhesive used. Indeed, an error in one of the bonding steps can compromise the restoration and its durability over time. Currently, adhesives can be classified into three categories: ER, SE, and UAs. The latter can be used according to the ER technique or the SE technique. All these developments have the aim of simplifying the procedures for the practitioner and limiting the risk of error responsible for subsequent failure.

Although SE adhesives have reduced the clinical time required for procedures, some HL degradation and poor etching capabilities have been reported. This can be explained by the hydrophilic nature of this type of adhesive [18,19,20,21,22]. These adhesives tend to leave residual water in the HL, which will lead to a decrease in the degree of conversion resulting from a drop in the dentinal mechanical properties [23,24,25]. In addition, the hydrophilic nature of the HL will play the role of a semi-permeable membrane that allows the movement of water [22,26] and increases the vulnerability of this layer to biomechanical degradation forces [21]. Studies have been carried out to try to solve this problem, including use of a prolonged drying time [27], drying with warm air [28,29], addition of a hydrophobic bonding agent [30,31], and/or performing a double layer technique [32]. All these studies have shown an initial increase in bond strength, but contain little information concerning the durability of this improved HL over time [33,34].

Intrinsically, the dentin substrate contains more water and less Hap than enamel. The prolonged application as well as rubbing of UAs can have a positive advantage on the infiltration of the functional monomers in the dentinal structure and a better evaporation of water as well as solvent. The result can be a more uniform adhesive layer and therefore obtaining a better bond [35,36,37].

In this study, a universal adhesive, PBU, was used to evaluate the bond strength of composite resin to dentin. The ER method was adopted for bonding, as recommended by the manufacturer. This is a sensitive technique that calls into play the expertise of the operator to obtain a slightly moist or wet state of dentin to ensure quality bonding. With this adhesive, the risk of error in this bonding technique has been reduced due to its ability to mix with water and form a uniform layer regardless of the state of the dentinal surface. Indeed, most universal hydrophobic adhesives tend to separate from water. In the presence of an excessively wet dentin surface, obtaining a uniform adhesive layer is almost impossible, leaving spaces that can weaken the bond and cause postoperative sensitivity. This adhesive can overcome the surface tension of water and is able to spread and form a uniform and homogeneous layer even in the presence of excess water on the dentin. When drying with air, the water and the solvent evaporate simultaneously to form a stable layer over the entire dentinal surface and thus ensure optimal dentinal adhesion [31,38].

This original study’s aim was to evaluate the adhesion strength of composite resins using a new device that allows the ejection of sterile water as well as a flow of pure air without humidity and other contaminants. It can also eject a disinfecting product such as chlorhexidine. This device is housed in a compact box that may be attached to any type of dental chair. Plus, the use of anhydrous copper sulfate makes it possible to highlight the humidity present in the air jet of dental chairs. Anhydrous copper sulfate, which is a white powder, turns blue when in contact with humidity and thus reveals the presence of water [39].

The results of this study showed that the SBS was significant between DENTIPURE and ADEC with *p* < 0.05. The significance of these results lies in the fact that this device can eject a flow of pure air without contamination with water or other impurities thanks to its unique design, using which the air duct is independent of that of water, thereby eliminating the risk of moisture mixing or incorporating with air as the adhesive dries. In fact, when talking about adhesive dentistry, the humidity in the oral cavity should be taken into consideration. This is due to the properties of adhesive systems and especially their performance, which can be affected in a negative way in contact with water, saliva, and blood [40,41,42], resulting in a decline in the bond strength of resin composite to dental substrate [43]. A previous study by Amsler et al. showed a decrease in bond strength following an increase in the humidity when using four types of adhesives [44]. The explanation for the aforementioned statement could rest with several causes, among which is humidity, that will prevent the complete evaporation of the solvent and lead to insufficient infiltration of the adhesive and therefore the formation of poor HL. In addition, humidity will prevent complete polymerization of the adhesive [41,45]. Furthermore, this unit is also equipped with an air pressure regulator. This allows for a uniform airflow throughout the drying process, promoting uniform spreading of the adhesive over the entire dentinal surface and thus avoiding excessive air pressure, which will result in discontinuous spreading or loss of adhesive.

The intermediate bond strength results obtained by the KAVO equipment could be explained by it having a more advanced air/water irrigation system than the ADEC. Indeed, the three-function syringe of the KAVO system has a new conceptual design that is different from the conventional one used in the ADEC and most dental units. According to the manufacturer, this design should avoid mixing humidity with air during function. In addition, it is equipped with an option that allows the practitioner to warm the air/water on a preselected level basis. As cited before, warm air can improve bonding to dentin [12]. The KAVO dental chair is equipped with an automated disinfection system. Near the sterile water is a bottle that can be filled with a disinfecting liquid (OXYGENAL 6) with a button that allows the flow of the product into all the water ducts of the dental chair. A specific protocol, including refilling the bottle, must be applied between patients and at the beginning of each week. This is done to prevent infection between patients.

UAs are composed of a mixture of acid functional monomers that are usually phosphoric acid esters, which have a higher pH than phosphoric acid [46]. These adhesives present a combination of hydrophilic and hydrophobic monomers, co-monomers, initiators, water, and solvent [47]. Several studies have been done to evaluate the effect of phosphoric acid etching of the dentin before the application of UAs and SE adhesives. Mixed results were achieved, from improved or decreased adhesion strength to no effect [48,49]. Etching the dentin prior to the application of UAs produces a thicker HL as well as deep monomer penetration, but the bond strength does not increase significantly [50]. Moreover, in vitro research on the bond strength of UAs did not find significant differences with or without prior dentin etching [51]. A study by Mohammed Q. Alqahtani (2015) [52] showed that the application of 35% orthophosphoric acid on dentin improves the bond strength of composite resins using SE and UAs. This could be explained by the presence of residual Hap from collagen fibrils, resulting in additional chemical adhesion and therefore an improvement in the bond strength on the etched dentin [53]. The other probable explanation is that the acidic monomers present in these adhesives can reach the basal part of the etched dentinal substrate when they are applied by making a shaking and rubbing motion. The result will be a better diffusion of the monomers and a better interaction with the underlying dentin, therefore better dentin adhesion [54].

It is important to note that the ER method is a sensitive technique and depends on the expertise of the operator. Excessive drying or wetting of the dentin can have negative effects on adhesion [55]. The introduction of the novel device tested in this research removes operator variability by making the step of adhesion to dentin repeatable between dentists in a standardized parameter (air pressure regulator).

It is crucial that an analysis of failure modes is performed to check if failure occurs at the adhesive interface. According to the findings obtained in this study, adhesive failure mode was dominant in all the tested specimens. These results are in accordance with those of a previous study [56] showing that higher adhesive failures were noted in the specimens with lower bond strength to dentin.

It should be noted that the new device used in this study (DENTIPURE KM) has a function that makes it possible to rinse the dentin with an antimicrobial agent (chlorhexidine) that can be administrated directly via the unit of the device. Accordingly, future research could be done by highlighting the impact of pure chlorhexidine ejected from this device. The limitations of the study reside in the use of extracted molars, one adhesive system, one bonding strategy, and the use of SBS instead of a microtensile bond strength test. Further clinical studies are needed to confirm these results. In addition, the use of other adhesive systems or another strategy could be implemented in future work. In this study, the samples were assessed by means of the SBS test due to the simplicity of preparing the specimens and not requiring the preparations of sticks of 1 mm^2^. A previous report suggested that the adhesive system is a significant factor for bond strength, irrespective of the testing method employed [57]. However, another study reported that the use of microtensile bond strength in this type of research is better than the SBS test [58]. Accordingly, in vitro and in vivo testing should be performed to confirm the current preliminary results. Moreover, it would be interesting to assess the surface characteristics of the resin–dentin interface (i.e., scanning electron microscopy) treated with different devices in future investigations. Further, subsequent studies could employ a precise, safe, and non-destructive technique such as scanning confocal microscopy for the evaluation of the resin–dentin interface [59]. Plus, longer-term evaluations are needed to assess the adhesive’s performance over extended periods, as clinical conditions may vary over time. Factors such as thermal cycling, occlusal forces, and exposure to saliva could influence bond strength and failure modes differently in vivo. Investigating variations in application protocols such as pretreatment with chemical agents including sodium hypochlorite or ethylenediaminetetraacetic acid [60], adhesive layer thickness, application time, or curing techniques could optimize bond strength and minimize failure rates, enhancing clinical outcomes. Addressing these limitations and exploring future perspectives will contribute to a comprehensive understanding of universal adhesive systems’ performance, ultimately enhancing their clinical applicability and patient outcomes.

## 5. Conclusions

In conclusion, while the DENTIPURE KM™ device shows promise in providing contaminant-free air during bonding, its impact on dentin bond strength compared to other devices like the KAVO™ appears minimal. Further research is needed to fully assess its potential in enhancing dentinal adhesion procedures.

## Figures and Tables

**Table 1:** Devices used in the study.

Devices	Manufacturer
DENTIPURE KM™	KM, Beirut, Lebanon
KAVO™ ESTETICA E30/E70/E80 Vision	KAVO, Biberach, Germany
ADEC™ A-dec Performer 200	ADEC, Newberg, OR, USA

**Table 2:** Study materials.

Material	Manufacturer	Main Components
Adhesive: Prime & Bond	DENTSPLY Charlotte, NC, USA	Dipentaerythritol penta acrylate monophosphate, polymerizeable dimethacrylate resin, polymerizeable PE trimethacrylate resin, diketon, organic phosphine oxide, stabilizers, cetylamine hydrofluoride, water.
Etching: Onyx	CENTRIX, Shelton, CT, USA	Phosphoric acid concentration: 40%
Bulk fill composite: SDR plus	DENTSPLY Charlotte, NC, USA	Urethane dimethacrylate resin, dimethacrylate resin, di-functional diluents barium and strontium, aluminofluorosilicate glasses, photo initiating system colorants.

**Table 3:** Mean and standard deviation of the shear bond strength in different groups tested in this study after 24 h aging.

Devices	PBU
ADEC	12.572 (5.901) ^a^
DENTIPURE KM	15.018 (4.895) ^b^
KAVO	13.681 (5.011) ^a,b^

^a,b^: Different letters indicate the presence of significant difference between groups according to Tukey post hoc test.

**Table 4:** Tukey’s multiple comparisons of the different groups tested in the present study.

Grups	N	1	2
ADEC	60	12.57211	
KAVO	60	13.68098	13.68098
DENTIPURE KM	60		15.01836
*p*		0.486	0.351

The group means of the homogeneous subsets are displayed.

## Data Availability

The data that supports the findings of this study are available upon reasonable request from the author (K.K).
